# Is Health Education among the Decisive Factors for the Diet Quality of Pregnant Women in Poland?

**DOI:** 10.3390/nu15112627

**Published:** 2023-06-04

**Authors:** Anna Demuth, Joanna Ratajczak, Urszula Czerniak, Katarzyna Antosiak-Cyrak

**Affiliations:** 1 Department of Anthropology and Biometry, Faculty of Sport Science, Poznan University of Physical Education, 61-871 Poznań, Poland; demuth@awf.poznan.pl (A.D.); czerniak@awf.poznan.pl (U.C.); 2 Department of Swimming and Water Rescue, Poznan University of Physical Education, 61-871 Poznań, Poland; antosiak-cyrak@awf.poznan.pl

**Keywords:** pregnancy, health education, diet quality, diet quality determinants

## Abstract

Health education (HE), an educational process that leads to increased nutritional awareness and improved health, is one of the factors influencing diet quality (DQ) during pregnancy. The aim was to evaluate the DQ of pregnant women and its determinants considering their HE. The study included 122 pregnant women aged 20–40 years. DQ was assessed using the Kom-PAN^®^ questionnaire and the Pro-Healthy Diet Index (pHDI). Data collected included dietary habits, socio-demographic data, education level, place of residence, and maternal lifestyle-related characteristics, namely, pre-pregnancy weight, trimester of pregnancy, and pre-pregnancy and pregnancy physical activity (PA). Weekly energy expenditure was determined using the Polish version of the PPAQ questionnaire. HE at school more than tripled the odds of a higher DQ. Women in their second trimester were 54% more likely to have a higher DQ than women in their third trimester of pregnancy. Undertaking pre-pregnancy PA increased the odds of a higher DQ 2.5 times. Comparative analyses performed in a group of women with HE (HEG, n = 33) and without HE (nHEG, n = 89) showed better DQ in the former, but this was still unsatisfactory in health-promoting properties. The results obtained showed that the HE and trimester of pregnancy and pre-pregnancy Pa influenced DQ in pregnant women.

## 1. Introduction

Health and dietary behaviors before and during pregnancy consistently remain an important and ongoing area of research [1,2,3]. Epidemiological studies have highlighted the significance of assessing diet quality and its determinants as the consequences of inadequate nutrition expose not only women but also their children to poorer health outcomes for the rest of their lives [4]. 

Among the factors influencing diet quality in pregnant women age, socioeconomic and lifestyle variables are the most commonly reported factors [5,6]. Other factors include pre-pregnancy BMI, physical activity, smoking, and alcohol consumption [7,8,9,10,11,12] but also nutritional knowledge, which has been reported to play an important role in pregnancy and influencing dietary choices. Obesity and overweight are currently a serious problem among women of reproductive age. The Central Statistical Office in Poland shows that the percentage of women of reproductive age (20–39 years) with excessive body weight (BMI > 25 kg/m^2^) increased from 25.8% to 31.3% between 2009 and 2019 [13]. Efforts to provide appropriate health education and care for pregnant women should be intensified due to the fact that almost one in three Polish women of reproductive age has problems with maintaining a healthy body weight.

Despite the proven link between maternal nutrition and pregnancy outcomes, many pregnant women do not follow the dietary recommendations. Moreover, behaviors such as a sedentary lifestyle and unhealthy eating habits are common among pregnant women worldwide, including Poland [14,15,16,17,18,19]. It has been indicated that such behaviors during pregnancy are caused by both non-adherence to the recommendations and insufficient health education and health promotion [20]. 

In Poland, health education is understood as a didactic and educational process in which pupils starting from primary school learn how to maintain and improve their own and other people’s health, how to create a health-favorable environment, and, in the case of illness or disability, how to actively participate in its treatment, cope with its negative effects, and reduce its consequences [21]. An important part of health education in schools is the development of appropriate eating behaviors, by which pupils acquire competences in the knowledge of basic nutrients and their role in the body, the preparation and storage of food, the knowledge of diseases related to poor nutrition, the knowledge of labeling food packages, and the ability to prepare menus for different groups of people [22]. Health education also plays an important role in shaping health-promoting attitudes by practicing hygienic behaviors that are safe for health, as well as the use of prevention, the practice of physical activity, and the consolidation of knowledge about its benefits. 

Researchers emphasize that the level of knowledge and awareness may influence the level of acceptance of educational messages, and, therefore, their effectiveness, which is why health education may be particularly important [23,24]. As schools play an important role in meeting the nutritional needs of children and adolescents and in shaping appropriate behavior [21], health education should already start at an early stage of education. Unfortunately, this type of education has not always been included in the compulsory school curriculum, Poland being an example. In Poland, health education was included in the core curriculum of general education for all types of schools only in 1997 [25]. However, it is not a separate school subject, but its content has been included in many subjects, e.g., biology, family life education, social studies, and safety education. An important step in the Polish education system was linking health education with physical education 2013. Since then, physical education has been playing a leading role in health education. According to the Education Law in Poland [26], a child’s compulsory education starts at the beginning of the school year in the calendar year in which the child turns 7 and lasts until the end of primary school, no longer than until the age of 18. Therefore, all people who started primary school in 1997 or later have achieved the expected learning outcomes for health education. In contrast, people who started school before 1997 did not receive health education classes at school, and their knowledge about health behavior comes from a variety of sources.

So far, little attention has been paid to the impact of early health education on diet quality in pregnant women. Considering the importance of diet in pregnant women and studies assessing diet quality, the aim of this study was to evaluate the diet quality of pregnant women and identify its determinants with particular attention to health education.

## 2. Materials and Methods

### 2.1. Survey Design and Sample

The survey was conducted in Poznań in free birthing schools, i.e., where access is universal, and there is no extra cost for parents to attend. Due to the lack of official data on the percentage of pregnant women attending antenatal classes, the sample size was estimated based on the list of women attending the classes in three randomly selected birthing schools between September and December 2019. The total number of the women attending antenatal classes was 170, all of whom were asked to participate in the study. Slovin’s formula (see below) was used to calculate the sample size with a 5% margin of error and 95% confidence interval [27]. The minimum number of the necessary sample size to meet the criteria listed above was 119. Of the total number of 170 women, 129 (75.9%) agreed to participate in the study, and 41 (24.1%) refused. In addition, seven of the women were excluded from the study due to incomplete questionnaire answers. Finally, 122 women were included in the study.

$$n=\frac{N}{1+Ne^{2}}$$

n—sample size; N—population size; e—margin error.

The study took the form of a direct, individual questionnaire survey. The participants completed the questionnaire on their own. In case of any problems with understanding the questions, the interviewer was helpful in explaining the inaccuracies. The collected information included eating habits, sociodemographic data, i.e., age, level of education (university/secondary/vocational/primary), presence of health education in school, place of residence (urban/rural), and maternal lifestyle-related characteristics, i.e., pre-pregnancy weight, trimester of pregnancy, and pre-pregnancy and pregnancy physical activity (PA). In addition, the respondents were asked about the year in which they started primary school. This made it possible to distinguish two groups of women: with health education (i.e., women who started primary school education after 1997; HEG; n = 33) and without health education (i.e., women who started primary school education before 1997; nHEG; n = 89). The distinguished groups were further used for comparative analyses. The study was approved by the Bioethics Committee of the Poznan University of Medical Sciences (reference no. 878/19, 12 September 2019). All women gave written consent to participating in the study. 

The KomPAN^®^ questionnaire provided data on eating habits and enabled the calculation of the Pro-Healthy Diet Index (pHDI), which gave information on diet quality [28]. The index was the sum of the daily intakes (times/day) of 10 food groups with potentially beneficial outcomes: 1. wholemeal bread; 2. grains and coarse-ground groats; 3. milk (including flavored milk, cocoa, coffee with milk); 4. fermented milk beverages; 5. curd; 6. white meat; 7. fish; 8. legumes; 9. fruits; and 10. vegetables. Each respondent reported habitual consumption of the above-mentioned products by indicating one of the six frequency categories: never, 1–3 times a month, once a week, a few times a week, once a day, and a few times a day. Those categories were converted to daily frequency expressed as times/day: never (0), 1–3 times a month (0.06), once a week (0.14), a few times a week (0.5), once a day (1.0), and a few times a day (2.0). The pHDI values ranged from 0 to 100 points and were calculated using the formula below. The pHDI values in the range of 0–33 points were defined as low, in the range of 34–66 points as moderate, and in the range of 67–100 points as high. The higher the value, the greater the intensity of health-promoting properties in the diet and, therefore, the better quality of the diet [28].

$$pHDI\;in\;points=\frac{100}{20}\times sum\;of\;the\;consumption\;of\;10\;food\;groups\;(times/day)$$

The Polish version of PPAQ questionnaire enabled us to determine the weekly energy expenditure (MET hour/week^−1^) [29]. The respondents self-assessed their physical activity levels by filling in a questionnaire consisting of 33 items grouped into the following activity categories: household/caregiving (15 items), occupational (5 items), sports/exercises (7–9 items), transportation (3 items), and inactivity (3 items). The declared duration of performance of particular tasks was assigned fixed numbers of minutes (0; 0.12; 0.50; 1.0; 2.0; 3.0) and then multiplied by the number of days of performance of the tasks per week. The obtained values were then multiplied by intensity (MET) in accordance with the guidelines in “Compendium of Physical Activities: an update of activity codes and MET intensities” [30], thus obtaining the energy expenditure measured in Metabolic Equivalent of Task (MET). The following levels of intensity were assigned to the different activities: sedentary < 1.5 METs; light 1.5–<3.0 METs; moderate ≥3.0–≤6.0 METs; and vigorous > 6.0 METs. In addition, the respondents were asked if they had undertaken physical activity before pregnancy. The participants could choose between yes/no answers.

### 2.2. Analysis

All statistical analyses were performed using STATISTICA 13 (Dell Inc.; Tulsa, OK, USA, StatSoft Polska, Cracow, Poland, 2017). The threshold of statistical significance was set at *p* ≤ 0.05. The distribution of the variables was tested using the Shapiro–Wilk test. For quantitative variables, arithmetic means and standard deviations (SD) were calculated. The median, lower, and upper quartiles were calculated for the frequency of consumption of 10 product groups. The Mann–Whitney (Z) test was used to test the significance of differences between the distinguished groups. The Chi-square test (χ^2^) was used for comparative analysis of categorical variables. The Spearman’s rank correlation coefficients (r) were used to assess the presence and strength of the associations between diet quality and consumption of selected food products, as well as sociodemographic data and maternal lifestyle-related variables. The interpretation of the correlation coefficients was as follows: weak (<0.3), moderate (0.3 to <0.5), strong (0.5 to <0.7), and very strong (≥0.7) correlation [31]. To identify the determinants of diet quality, multiple regression models were run with diet quality as the dependent variable. Only factors that were significantly correlated with diet quality were included in the models. Logistic regression analysis was used to assess the odds of having a higher-quality diet. The dependent variable was diet quality as assessed by the Pro-Healthy Diet Index (pHDI). The categorization of the two groups for the dependent variable in the logistic regression was based on pHDI values. Values ≤ 33 points were assigned to the “lower quality diet” category, whereas values > 33 points were assigned to the “higher quality diet” category. Odds ratios (ORs) and 95% confidence intervals (95% CI) were calculated.

## 3. Results

### 3.1. Group Characteristics

The characteristics of the participants are shown in Table 1. The mean age was 27.7 ± 3.7 years. The women from the health education group were younger than women in the group without health education (23.4 ± 1.5 vs. 29.3 ± 2.9; Z = −8.45; *p* < 0.001). The percentage of the women with a higher level of education was greater in nHEG than in HEG (76.4% vs. 54.6%; χ^2^ = 6.58; *p* = 0.037). The vast majority of participants had a higher level of education (70.5%) and lived in urban areas (68.9%). Of the participants, 49.2% were in their third trimester of pregnancy. The mean pre-pregnancy weight was 66.3 ± 14.3 kg. Undertaking physical activity before pregnancy was declared by 58.2% of the respondents. An assessment of the physical activity levels of the pregnant women showed that the highest energy expenditure was recorded for light and moderate intensity efforts ($\overset{-}{x}=$71.7 MET hour/week; $\overset{-}{x}=$72.9 MET hour/week), accounting for 37.5% and 38.2% of total physical activity, respectively.

### 3.2. Diet Characteristics

In the entire study group, the mean value of the pHDI was 26.3 ± 13.0 points. The women with health education had a higher value of the pHDI than the women without health education (HEG = 28.3 ± 12.7 points vs. nHEG = 20.9 ± 12.3 points; Z = 2.99; *p* = 0.002). There were no women with a high-quality diet in the whole study group; however, a moderate-quality diet was noted in 30.3% of the participants. 

In general, fruit and vegetables were consumed with the greatest frequency (on average once a day), whereas fish and legumes were consumed least frequently (on average 1–3 times a month). The remaining products were consumed with an average frequency of once to several times a week (see Supplement Table S1). A comparative analysis showed significant differences in the frequency of consumption of wholemeal bread (Z = 2.72; *p* = 0.007), grains and coarse-ground groats (Z = 2.43; *p* = 0.02), legumes (Z = 1.97; *p* = 0.049), and fruits (Z = 2.21; *p* = 0.03). Each time, the nHEG group was characterized by a lower frequency of consumption of the above-mentioned products.

The correlations of health education with the pHDI and ten food products with beneficial health outcomes are shown in Table 2. Positive and significant correlations were found for all variables except milk consumption, fermented milk beverages, curd, and white meat.

### 3.3. Food Determinants of Diet Quality 

Before testing the hypothesis concerning the food correlates of diet quality, the correlations between diet quality as the dependent variable and ten food products with a potentially beneficial effects on health were analyzed (Table 3). In each group, moderate but significant, strong, and very strong correlations between diet quality and the mentioned independent variables were found. In the group with health education, all variables were positively correlated with the diet quality. They were as follows: wholemeal bread (r = 0.58; *p* ≤ 0.001); grains and coarse-ground groats (r = 0.74; *p* ≤ 0.001); milk (r = 0.60; *p* ≤ 0.001); fermented milk beverages (r = 0.62; *p* ≤ 0.001); curd (r = 0.62; *p* ≤ 0.001); white meat (r = 0.35; *p* = 0.001); fish (r = 0.45; *p* ≤ 0.001); legumes (r = 0.47; *p* ≤ 0.001); fruits (r = 0.75; *p* ≤ 0.001); and vegetables (r = 0.81; *p* ≤ 0.001). In a group with no health education, no correlation was found for milk and white meat, whereas positive correlations for other food products were as follows: wholemeal bread (r = 0.50 *p* = 0.002); grains and coarse-ground groats (r = 0.42; *p* = 0.009); fermented milk beverages (r = 0.60; *p* ≤ 0.001); curd (r = 0.37; *p* = 0.02); fish (r = 0.33; *p* = 0.041); legumes (r = 0.53; *p* ≤ 0.001); fruits (r = 0.61; *p* ≤ 0.001); and vegetables (r = 0.73; *p* ≤ 0.001).

These significant variables were then included in the multiple regression model in order to assess which of them contributed most to explaining the variability in the diet quality in separate groups. According to the results obtained (Table 4), diet quality in the group with health education was determined by eight variables, i.e., vegetables, fermented milk beverages, milk, wholemeal bread, fruits, grains, coarse-ground groats, curd, and white meat. The model was significant and explained 99.6% of the variance in the diet quality F(8.75) = 2297.2; *p* ≤ 0.001). The consumption of vegetables (R^2^ = 0.607; *p* ≤ 0.001) and fermented dairy drinks (∆R^2^ = 0.223; *p* ≤ 0.001) made the greatest contribution to the prediction of the dependent variable.

In the group with no health education, six variables were included in the final diet quality model, i.e., vegetables, fermented milk beverages, grains and coarse-ground groats, fruits, legumes, and wholemeal bread. The model was significant and explained 93.9% of the variance in the dependent variable (F(6.31) = 79.5, *p* ≤ 0.001). As in the previous model, vegetables and fermented dairy drinks had the largest contribution to the prediction of the dependent variable (respectively: R^2^ = 0.594; *p* ≤ 0.001; ∆R^2^ = 0.179; *p* ≤ 0.001). The smallest, however still significant, contribution to explaining the variability of diet quality was made by wholemeal bread (∆R^2^ = 0.038; *p* ≤ 0.001).

### 3.4. Sociodemographic and Maternal Lifestyle-Related Determinants of Diet Quality 

The first step in assessing the sociodemographic and maternal determinants of diet quality was to examine the correlations between diet quality as the dependent variable and all the variables listed in Table 1. Significant correlations were found for variables such as age (r = 0.20; *p* = 0.026), health education (r = 0.27; *p* = 0.002), educational level (r = 0.20; *p* = 0.025), trimester of pregnancy (r = 0.31; *p* ≤ 0.001), pre-pregnancy PA (r = 0.26, *p* = 0.003), moderate PA (r = 0.19; *p* = 0.039), and vigorous PA (r = 0.19; *p* = 0.035). A regression model was then run with diet quality as the dependent variable. According to the results obtained (Table 5), diet quality was predicted by four variables, i.e., health education, trimester of pregnancy, moderate PA, and pre-pregnancy PA. The greatest contribution to the prediction of the dependent variable was made by health education (∆R^2^ = 0.069; *p* = 0.003), followed by the trimester of pregnancy (∆R^2^ = 0.063; *p* = 0.028). Then, moderate PA was added (∆R^2^ = 0.044; *p* = 0.013), and, in the last step, pre-pregnancy PA was included (∆R^2^ = 0.032; *p* = 0.032). The final model was significant and explained 20.8% of the variance of the diet quality (F(4.17) = 7.69; *p* ≤ 0.001).

### 3.5. The Odds Ratio of Higher-Quality Diet

**Figure 1:**
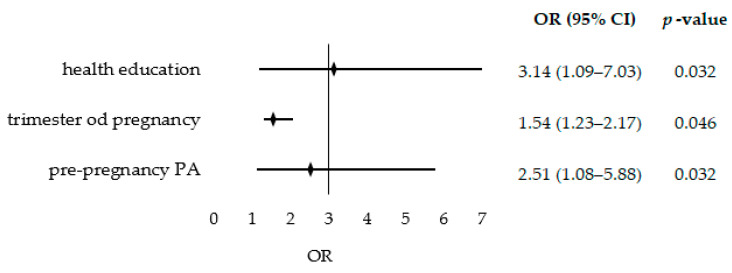
The odds ratio of a higher-quality diet.

A logistic regression analysis was performed to assess how the sociodemographic and maternal lifestyle-related predictors from Table 5 affected the odds of achieving a higher-quality diet (Figure 1). Unfortunately, due to lack of standards and cut-off points, a similar analysis could not be performed for moderate PA during pregnancy. The results showed that the presence of health education in the educational history of the surveyed participants more than tripled the odds of a higher-quality diet (OR = 3.14; 95% CI: 1.09–7.03; *p* = 0.032). The women in their second trimester were 54% more likely to have a higher-quality diet than the women in their third trimester of pregnancy (OR = 1.54; 95% CI: 1.23–2.17; *p* = 0.046). Undertaking PA before pregnancy increased the odds of a higher-quality diet by 2.5 times (OR = 2.51; 95% CI: 1.08–5.88; *p* = 0.032).

## 4. Discussion

The literature indicates that pregnancy is an important time in a woman’s life, contributing to changes in both her dietary habits and other health-related behaviors that are undertaken out of concern for her life and health and that of her baby [1,3,32]. Previous studies have shown a wide variation in the determinants of diet quality among pregnant women. In addition to social and cultural factors [24,33,34,35,36], nutritional knowledge and health education have also been indicated as factors influencing diet quality in pregnancy [35]. Therefore, the aim of this study was to assess the dietary quality of pregnant women and its determinants, with attention to health education as possible one.

Among the most commonly reported factors influencing the quality of pregnant women’s diets are age and socioeconomic variables, including the education level, which is considered to be an awareness variable that significantly influences dietary decisions [8,9,12,37,38,39,40]. In turn, the presented study highlighted the particularly important role of health education, trimester of pregnancy, moderate PA, and pre-pregnancy PA in shaping dietary habits and diet quality. According to the literature, younger mothers have poorer diet quality because they have lower levels of education, lower socioeconomic status, and less life experience, unlike older women [1,37,41,42,43,44,45,46,47]. However, our own results show that women in the no health education group, despite being older and having achieved a university degree, had poorer diet quality than younger women without a higher education but with health education in the core curriculum. This suggests that diet quality does not depend as much on age and educational attainment but, to a large extent, on the health education provided as part of compulsory schooling for children up to the age of 18. Our further analysis showed that participation in compulsory health education more than tripled the odds of having a better diet. Sedentary lifestyles and unhealthy eating habits are known to be common among pregnant women [17], but our results show that women with healthier pre-pregnancy behaviors were also those with better diets during pregnancy. In contrast to McGowan and McAuliffe [48], our study showed a significant positive influence of pre-pregnancy and pregnancy PA on diet quality, with the pre-pregnancy PA increasing the odds of a higher-quality diet during pregnancy by a factor of 2.5. This confirms that physical activity is an important target for nutrition and health interventions. In the presented study, women in the second trimester of pregnancy had a healthier dietary profile than women in the third trimester. This is on the contrary to Fernández-Gómez et al. [49], but consistent with McGowan and McAuliffe [42], who reported the odds in predicting the likelihood of following a healthy dietary pattern in each trimester. In their study, higher levels of maternal education together with normal maternal BMI as well as the nationality were important predictors of following a healthy diet in the second trimester. This indicates that women with higher levels of education also are more likely to make positive changes in their diet. Although awareness of the positive effects of a healthy diet and physical activity on pregnancy outcomes has been reported to be a strong motivator for changing dietary behaviors [50,51], it is not always sufficient to maintain changes until the end of pregnancy. As shown by McGowan and McAuliffe [48], 69 out of 95 women continued the healthy dietary pattern into the third trimester. Therefore, there is a strong need for research to investigate the reasons why healthy dietary behaviors are not maintained during pregnancy. 

A positive contribution of health education to dietary behaviors was also shown in the case of the Pro-Healthy Diet Index, which provides information on diet quality. The diets of women who received counselling and education on healthy eating and lifestyles were of better quality than those of women who did not receive adequate substantive support. In addition, health education was positively associated with the intake of wholemeal bread, grains and coarse-ground groats, fish, legumes, fruit, and vegetables but not with intakes of milk, fermented milk beverages, curd, or white meat. Our results differ from those obtained by Goodarzi-Khoigani et al. [52], who showed that health education was positively associated with the intake of vegetables and fish but not bread, legumes, dairy products, or fruit in the Japanese population. 

Unfortunately, despite the positive contribution of health education to dietary behaviors and noticeable differences in the level of DQ and the frequency of consumption of selected groups of products, the diets of women with nutrition education were not in accordance with nutritional recommendations [53,54]. In the surveyed groups, the consumption of products with beneficial health effects was insufficient, which corresponds with the findings of other authors [14,16,18]. In general, the respondents consumed fruit and vegetables most frequently (once a day on average), which is significant, as they are the basis of a healthy diet in many nutritional recommendations, mainly because of the vitamins, minerals, and antioxidants they contain [55,56]. The remaining food products were consumed with an unsatisfactory frequency, and the identified dietary errors were particularly related to insufficient consumption of whole grain products (wholemeal bread, groats, oatmeal), fish, and legumes. Given the fact that whole grain products are a good source of fiber and have a positive impact on the prebiotic index [57,58], a well-balanced diet should be rich in these products. Unfortunately, only 16% of the women with health education met the recommendations of several servings of whole grain per day [57], compared to 9% of the women without health education. The recommended intake of 2–3 portions of fish per week was reported by 10% of the women with health education and only 3% of those without health education. The consumption of legumes was also low. However, this can be regarded as a positive outcome, especially if they had been eaten as raw sprouts (e.g., beansprouts). Similar to legumes, sprouts are a good source of protein [59] and also of health-maintaining nutrients such as glucosinolates, phenolics, and isoflavones [60]. However, it should be noted that sprouts also belong to a group with a high risk of Listeria monocytogenes infection [61], and, unlike maternal listeriosis infection, fetal or neonatal infection carries a high risk of fatal complications [62]. Therefore, pregnant women should limit their consumption of sprouts. 

The results obtained indicate the positive impact of educational programs conducted in Polish schools aimed at implementing the principles of proper nutrition described by the healthy eating pyramid [54]. In the group of the women with health education, eight out of ten groups of products with potentially health-promoting properties determined the quality of the diet (i.e., vegetables, fermented milk beverages, milk, wholemeal bread, fruit, grains and coarse-ground groats, curd, and white meat). In turn, in the group without health education, the variety of food determinants of diet quality was smaller. Only six out of ten recommended products explained the variance in diet quality, i.e., vegetables, fermented milk beverages, grains and coarse-ground groats, fruit, legumes, and wholemeal bread. However, it should also be noted that the consumption of vegetables and fermented dairy drinks was one of the determining factors of diet quality in each studied group. 

Previous studies have shown that women’s compliance with recommendations increased when they were given detailed explanations on the importance of the recommended food products [23,24]. On the other hand, the lack of adequate knowledge about nutritional recommendations of those responsible for developing nutritional awareness have been identified as one of the barriers to changing dietary behaviors [63,64,65]. Therefore, it is possible that the results obtained in the present study are caused by inadequate health education in Polish schools, e.g., the content provided may be insufficient or not adapted to the age of the recipients, yet the individual non-adherence to the recommendations cannot be excluded. 

Important clues for nutrition education also come from studies that indicate the preferred form of knowledge transfer. As was shown by Wise and Arcamone [66], among adolescents, the best way to learn about nutrition was to listen to teachers and health professionals. Unfortunately, it is not appreciated in Poland. Here, health education is provided only by schoolteachers, nor is it not a separate school subject, but it is implemented in a number of different subjects in the form of selected individual class topics. Crucially, partners and relatives are an important source of nutritional support for mothers and mothers-to-be [67]; in order to improve the quality of pregnant women’s diets, it is also necessary to educate and increase knowledge about the positive or reinforcing effects of healthy nutrition also in the woman’s immediate environment.

### Limitations

This study has some strengths and limitations. The study included a group of women attending childbirth school, and access to health education was taken into account. The KomPAN^®^ and PPAQ (Polish version) questionnaires used in the study have good relevance, and acceptable test–retest reliability of the test–retest, therefore, represent a reliable set of data. The PPAQ questionnaire has been adapted to the cultural conditions of many countries, including Poland, allowing international comparisons to be made regarding the level of AP of pregnant women. Furthermore, the study was conducted in the form of a direct questionnaire interview, which allowed us to better understand the questions and obtain more complete and reliable information about the dietary habits of the women surveyed. However, we are aware of some limitations. It was a cross-sectional study, in which diet quality was analyzed based on questions about general food consumption rather than questions about specific dietary components. Future research should include this type of data to gain full insight into the complex model of determinants of dietary quality. The Pro-Healthy Diet Index (pHDI) used to assess diet quality is based on the consumption of health-promoting products recommended in the Mediterranean diet and included in the healthy eating pyramid.

## 5. Conclusions

The present study highlighted the particularly important role of health education, trimester of pregnancy, moderate PA, and pre-pregnancy PA in shaping dietary habits and diet quality. We recommend that the proposed interventions for the nutritional education of women of reproductive age include not only nutritional aspects but also physical activity adapted to the gestational age and capabilities of the pregnant women. Appropriate adaptation of the interventions to the individual needs of the woman, her preferences, and, above all, her knowledge and health habits can effectively influence the modification of her dietary behavior during pregnancy. The present study also has practical implications. The results obtained can be used by institutions providing health education to preconceptional and pregnant women to develop an appropriate strategy aimed at raising awareness of the importance of proper nutrition during pregnancy and possibly changing inappropriate eating habits.

## Figures and Tables

**Table 1:** Characteristics of surveyed group.

Variables	Total n = 122	HEG n = 33	nHEG n = 89	*p*-Value
age (years)	27.7 ± 3.7	23.4 ± 1.5	29.3 ± 2.9	<0.001
educational level (%) (n)				
- primary	5.7 (7)	12.1 (4)	3.4 (3)	0.037
- vocational/secondary	23.8 (29)	33.3 (11)	20.2 (18)	
- university	70.5 (86)	54.6 (18)	76.4 (68)	
place of residence (%) (n)				
- rural	31.1 (38)	33.3 (11)	30.3 (27)	0.751
- urban	68.9 (84)	66.7 (22)	69.7 (62)	
trimester of pregnancy (%) (n)				
- I	9.8 (12)	18.2 (6)	6.7 (6)	0.161
- II	41.0 (50)	39.4 (13)	41.6 (37)	
- III	49.2 (60)	42.4 (14)	51.7 (46)	
pre-pregnancy weight (kg) (mean± SD)	66.3 ± 14.3	63.5 ± 13.8	67.3 ± 14.4	0.110
pre-pregnancy PA (%) (n)				
- no	41.8 (51)	54.5 (18)	37.1 (33)	0.082
- yes	58.2 (71)	45.5 (15)	62.9 (56)	
pregnancy PA (METs; mean ± SD)				
- total PA	191.0 ± 118.7	187.6 ± 156.8	192.4 ± 102.1	0.177
- sedentary (<1.5)	41.1 ± 31.6	41.2 ± 31.7	41.1 ± 31.5	0.977
- light PA (1.5–<3.0)	71.7 ± 38.9	69.2 ± 42.5	72.6 ± 37.6	0.536
- moderate PA (≥3.0–≥6.0)	72.9 ± 75.8	68.6 ± 92.8	74.6 ± 68.9	0.132
- vigorous PA (>6.0)	5.3 ± 22.3	8.6 ± 33.7	4.1 ± 16.2	0.762

HEG—group with health education; nHEG—group without health education; *p* ≤ 0.05—a statistically significant value.

**Table 2:** Correlation coefficients of the health education with the pHDI and 10 food products with beneficial health outcomes.

	r	*p*-Value
1. pHDI	0.27	0.002
2. wholemeal bread	0.25	0.005
3. grains and coarse-ground groats	0.23	0.012
4. milk	0.10	0.269
5. fermented milk beverages	0.14	0.111
6. curd	0.16	0.082
7. white meat	0.14	0.112
8. fish	0.19	0.039
9. legumes	0.19	0.033
10. fruits	0.21	0.019
11. vegetables	0.18	0.044

*p* ≤ 0.05—a statistically significant value.

**Table 3:** Food correlates of diet quality in surveyed groups.

Variables	HEG	nHEG
wholemeal bread	0.58 *	0.50 *
2. grains and coarse-ground groats	0.74 *	0.42 *
3. milk	0.60 *	0.26
4. fermented milk beverages	0.62 *	0.60 *
5. curd	0.62 *	0.37 *
6. white meat	0.35 *	0.28
7. fish	0.45 *	0.33 *
8. legumes	0.47 *	0.53 *
9. fruits	0.75 *	0.61 *
10. vegetables	0.81 *	0.73 *

HEG—group with health education; nHEG—group without health education. * a statistically significant correlation coefficient.

**Table 4:** Regression analysis of food determinants of diet quality in distinguished groups.

Variables	R^2^	β	F	*p*-Value
Model 1: HEG	0.996		2297.2	<0.001
vegetables		0.28		<0.001
fermented milk beverages		0.16		<0.001
milk		0.23		<0.001
wholemeal bread		0.20		<0.001
fruits		0.22		<0.001
grains and coarse-ground groats		0.20		<0.001
curd		0.16		<0.001
white meat		0.06		<0.001
Model 2: nHEG	0.939		79.5	<0.001
vegetables		0.21		0.004
fermented milk beverages		0.34		<0.001
grains and coarse-ground groats		0.16		0.002
fruits		0.38		<0.001
legumes		0.25		<0.001
wholemeal bread		0.26		<0.001

*p* ≤ 0.05—a statistically significant value.

**Table 5:** Regression analysis of socio-demographic and maternal lifestyle-related determinants of diet quality.

Variable	R^2^	β	F	*p* Value
	0.208		7.69	<0.001
health education		0.25		0.003
trimester of pregnancy		0.21		0.028
moderate PA		0.19		0.013
pre-pregnancy PA		0.18		0.032

*p* ≤ 0.05—a statistically significant value.

## Data Availability

Data are available on request from the corresponding author.

## Supplementary Materials

The following supporting information can be downloaded at: https://www.mdpi.com/article/10.3390/nu15112627/s1, Table S1. Dietary characteristics of surveyed women.
