# Supplementary material for: Is Health Education among the Decisive Factors for the Diet Quality of Pregnant Women in Poland?
Source: Nutrients. 2023 Jun 4;15(11):2627. doi: 10.3390/nu15112627 (PMC10255733; doi:10.3390/nu15112627)
Supplement: Supplementary file 1 [file nutrients-15-02627-s001.zip › nutrients-2397907-supplementary.pdf]

Table S1. Dietary characteristics of surveyed women

	1	2	3	4	5	6	7	8	9	10
**TOTAL**										
Median	0.14	0.14	0.50	0.50	0.14	0.50	0.06	0.06	1.00	1.00
Q_1	0.06	0.06	0.14	0.14	0.06	0.14	0.06	0.06	0.50	0.50
Q_3	0.50	0.50	1.00	0.50	0.50	0.50	0.14	0.14	2.00	2.00
**HEG**										
Median	0.50	0.14	0.50	0.50	0.50	0.50	0.14	0.06	1.00	1.00
Q_1	0.06	0.06	0.14	0.14	0.06	0.14	0.06	0.06	0.50	0.50
Q_3	0.50	0.50	1.00	0.50	0.50	0.50	0.14	0.14	2.00	2.00
**nHEG**										
Median	0.06	0.06	0.50	0.50	0.14	0.50	0.06	0.06	0.50	0.50
Q_1	0.00	0.00	0.14	0.14	0.06	0.14	0.06	0.00	0.50	0.50
Q_3	0.50	0.50	1.00	0.50	0.50	0.50	0.14	0.06	1.00	2.00

HEG - group of women with health education, nHEG - group of women without health education
1. wholemeal bread; 2. grains and coarse-ground groats; 3. milk; 4. fermented milk beverages; 5. curd; 6. white meat; 7. fish; 8. legumes; 9. fruits; 10. vegetables

0.00 – never; 0.06 – 1-3 times a month; 0.14 – once a week; 0.50 – a few times a week; 1 – once a day; 2 – a few times a day
